# Multilocus Gene Analyses Indicate *Tamarix aphylla* as Reservoir Host of Diverse Phytoplasmas Associated with Witches’ Broom and Yellowing Symptomatology

**DOI:** 10.3390/plants13091248

**Published:** 2024-04-30

**Authors:** Seyyed Alireza Esmaeilzadeh-Hosseini, Ghobad Babaei, Francesco Pacini, Assunta Bertaccini

**Affiliations:** 1 Plant Protection Research Department, Yazd Agricultural and Natural Resources Research and Education Centre, Agricultural Research, Education and Extension Organization (AREEO), Yazd, Iran; 2 Plant Protection Research Department, Chaharmahal and Bakhtiari Agricultural and Natural Resources Research and Education Centre, Agricultural Research, Education and Extension Organization (AREEO), Shahrekord, Iran; ghobad.babaee@gmail.com; 3 Department of Agricultural and Food Sciences, Alma Mater Studiorum—University of Bologna, 20127 Bologna, Italy; francesco.pacini2@unibo.it

**Keywords:** ‘*Candidatus* Phytoplasma asteris’, ‘*Ca*. P. australasiae=australasiaticum’, ‘*Ca*. P. trifolii’ disease, sequencing, 16S rRNA gene, *tuf* gene, *secA* gene

## Abstract

Tamarisk witches’ broom, yellowing, and little leaf symptoms were observed during 2018–2023 surveys of rural deserts in central regions of Iran with the highest disease incidence up to 72% in Chah Afzal (Yazd province). A verification of the presence and identity of phytoplasmas associated with these symptoms was then performed. Tamarisk tree branch cuttings obtained from symptomatic plants sprouted up to 90.3% but with 15–25 days’ delay compared to the asymptomatic ones and showed internode shortening and witches’ broom, while the branch cuttings from asymptomatic plants had normal growth and sprouted up to 97.8%. Phytoplasma transmission by dodder bridges to periwinkle did not succeed, while nested polymerase chain reaction on the phytoplasma ribosomal gene followed by RFLP and phylogenetic analyses revealed the presence of ‘*Candidatus* Phytoplasma asteris’, ‘*Ca*. P. australasiae=australasiaticum’, and ‘*Ca*. P. trifolii’ (ribosomal subgroups 16SrI-B, 16SrII-D, and 16SrVI-A, respectively) in the samples from symptomatic plants only. Further amplifications were performed on selected phytoplasma-positive samples on *tuf* and *secA* genes, and the produced sequences indicated the presence of mixed phytoplasma infection in some of the samples. In particular, in the *tuf* gene, a mixed infection of ‘*Ca*. P. australasiae=australasiaticum’ and ‘*Ca*. P. trifolii’ was detected, while in the *secA* gene, the presence of ‘*Ca*. P. asteris’ or ‘*Ca*. P. tritici’ strains was identified. The first-time detection of diverse phytoplasma strains in symptomatic *T. aphylla* suggests that this species represent a relevant source of infection for the agricultural crops and for landscape plants especially when temperature allows insect vector transmission, and therefore, it represents a risk in every environment especially in the frame of climatic changes.

## 1. Introduction

The genus *Tamarix* (tamarisk, salt cedar, taray) is composed of about 50–60 species of flowering plants of the family *Tamaricaceae*, native to dry areas of Eurasia and Africa. In Iran, tamarisk trees [*Tamarix aphylla* (L.) Karst.] have been planted in several rural areas especially near the fields as forestry for anti-desertification. Since the species is very resistant to saline and alkaline soils, it is also used as a windbreak, shade tree in agricultural fields of dry areas and as a barrier to fire. Phytoplasmas are cell wall-less bacteria that belong to the class *Mollicutes* and recently their presence was detected in *T. aphylla* plants showing witches’ broom symptoms [1]. They are bacteria inhabiting the phloem tissues of more than 1000 different plant species [2,3] and are transmitted mostly by leaf- and planthoppers [4]. Based on the restriction fragment length polymorphism (RFLP) and sequence analysis of the 16S rRNA gene, phytoplasmas are classified in ‘*Candidatus* Phytoplasma’ species and in ribosomal groups and subgroups [5,6,7]. Different phytoplasmas infect agricultural, forest, and a range of plant species worldwide; therefore, it is important to know their identity to be able to verify the presence of specific insect vectors and alternative host plants. Since *T. aphylla* is used as a wind breaking or reforestation species in several agriculturally and environmentally relevant areas of Iran where phytoplasma-associated diseases are usually present, the species could play a role in their epidemiological spreading. However, since there is no information about the *T. aphylla* phytoplasma-associated disease epidemiology and spreading, the objective of the present study was the molecular identification of phytoplasmas present in *T. aphylla* to clarify the possible role of the species as a reservoir for phytoplasmas associated with diseases in crops in agriculturally and environmentally relevant areas in Iran.

## 2. Results

**Figure 1:**
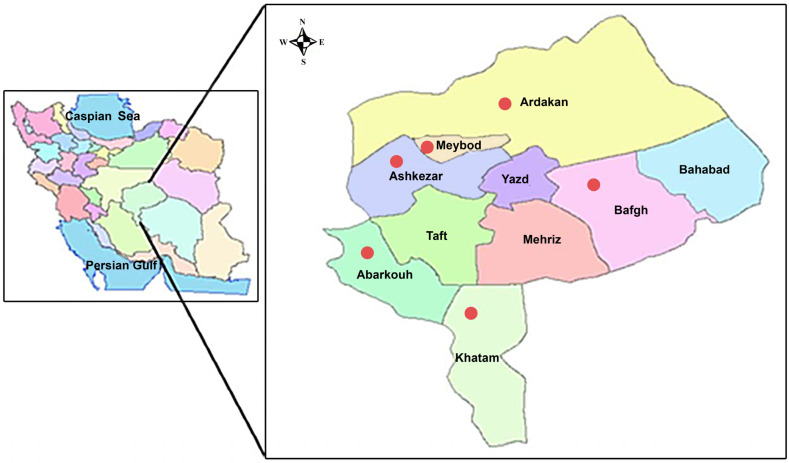
Map of the sampling areas (in different colors) in Yazd province, Iran.

**Figure 2:**
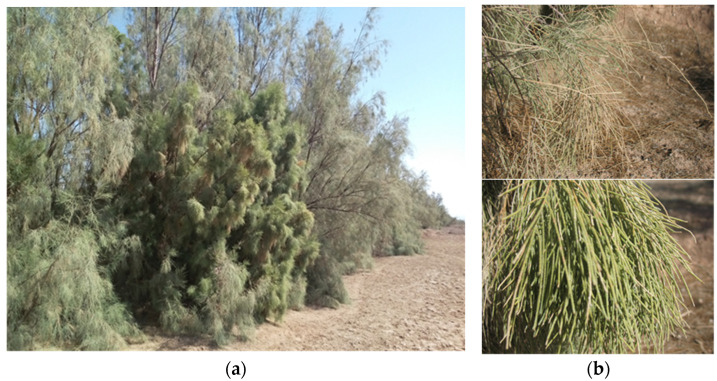
Witches’ broom, yellowing, and severe proliferation in *Tamarix aphylla* affected by the phytoplasma disease (**a**). Healthy branch (top) compared to infected one (bottom) (**b**).

During 2018–2023, the presence of *T. aphylla* witches’ broom and yellowing was surveyed in planted tamarisk trees in the rural desert of central regions of Iran, including Ardakan, Meybod, Bafgh, Herat, Ashkezar, and Abarkouh areas (Yazd province, center of Iran) (Figure 1). Symptoms of witches’ broom, yellowing, and little leaf were observed only in Chah Afzal and Mileshbar areas in Ardakan and Bafruiyeh area in Meybod (Figure 2). The highest disease incidence was 72% in planted *T. aphylla* forestry trees of Chah Afzal.

**Figure 3:**
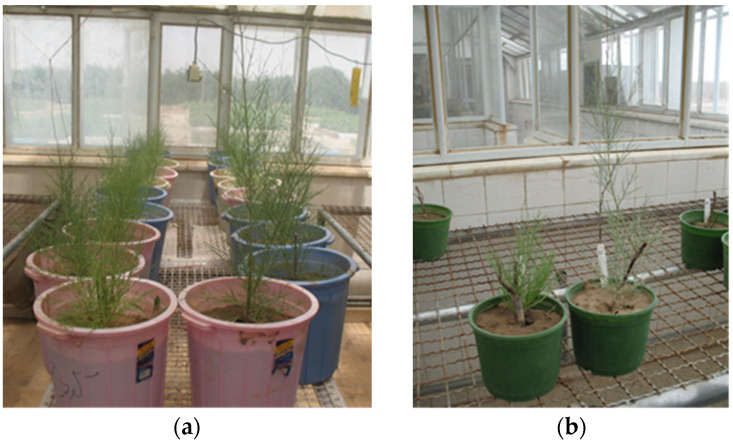
In both pictures (**a**,**b**), the left part of the picture is cuttings of diseased plant and right part of the picture is cuttings from healthy plant: in (**a**), a row of cuttings from diseased plant compared to a row of cuttings from healthy plant; and in (**b**), the pot in the left contains a cutting from a diseased plant, and the pot in the right contains a cutting from a healthy plant.

Tamarisk potted cuttings from asymptomatic and symptomatic plants sprouted 97.8% and 90.3%, respectively. Cuttings from symptomatic plants sprouted with 15–25 days’ delay compared to those from asymptomatic plants and showed internode shortening and witches’ broom. After four months, the average heights of the rooted cuttings were 74.2 and 31.2 cm for asymptomatic and symptomatic plants, respectively (Figure 3). The attempts of transmission of phytoplasmas via dodder to periwinkle failed. After 24 months, dodder-inoculated periwinkles did not have any phytoplasma symptoms, and the nested PCR results were negative.

**Figure 4:**
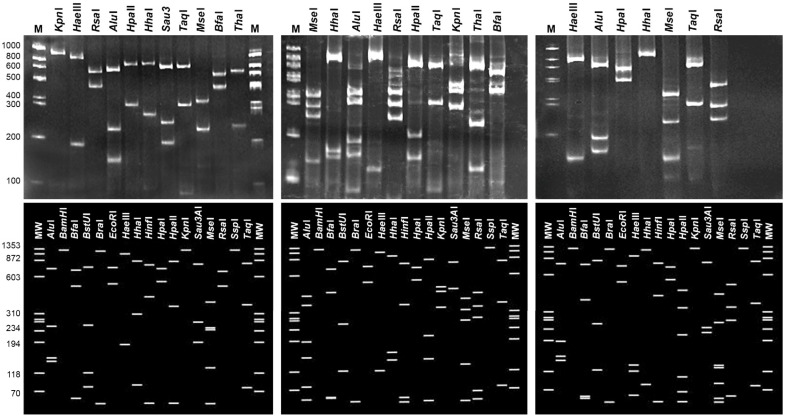
Real (top lane) and virtual (bottom lane) RFLP patterns [7] of 1.2 kb profiles of 16S rDNA amplicons obtained in nested PCR primed by P1/P7 and R16F2n/R16R2 and their sequences from phytoplasma strains referable to 16SrVI, 16SrI, and 16SrII, respectively. Lane M and MW 100 bp and phiX174 DNA/*BsuR*I (*Hae*III) DNA ladders. DNA products were digested with the enzymes listed at the top of the figures.

Amplicons of around 1.8, 1.4, and 1.2 kbp were obtained by direct and nested PCR on 16S rRNA gene from symptomatic tamarisk plants and from their branch cuttings. The nested PCR results were negative for asymptomatic plants and from their cuttings. Based on the RFLP patterns of partial 16S ribosomal sequences compared with those published for 16S rDNA amplicons amplified with the same primers [5], phytoplasmas referable to three ribosomal groups, 16SrI, 16SrII, and 16SrVI, were identified (Figure 4). 

From R16mF2/R16mR2 primed PCR products, six samples from each phytoplasma ribosomal group were sequenced and showed 100% sequence identity to each other. The sequence of one sample from each ribosomal group was deposited in GenBank under the accession numbers MK313753, MK313752, and MK313754, for 16SrI, 16SrII, and 16SrVI groups, respectively. Results of virtual RFLP analyses of the 1.2 kbp 16S rDNA of these sequences using *i*PhyClassifier showed patterns identical to 16SrI-B, 16SrII-D, and 16SrVI-A phytoplasma subgroups (Figure 4).

**Figure 5:**
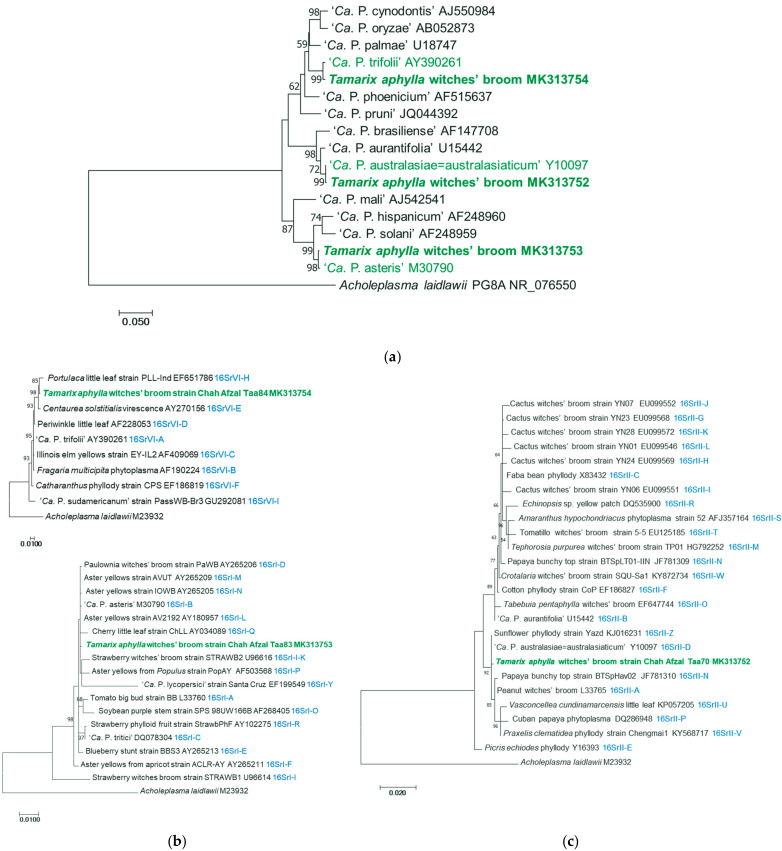
Molecular phylogenetic analysis by Maximum Likelihood method of the R16F2n/R16R2 sequence of 16S rRNA gene of *T. aphylla* phytoplasmas (in bold green) with ‘*Ca*. Phytoplasma’ (**a**) and phytoplasma strains in the 16SrVI (top) and 16SrI (bottom) (**b**) and 16SrII (**c**) subgroups; *Acholeplasma laidlawii* is used as an outgroup. Numbers at the nodes are bootstrap values based on 1000 repetitions. GenBank accession numbers for sequences are given following the phytoplasma and strain names, in light blue ribosomal subgroup affiliation, in light green strains of the *T. aphylla* phytoplasmas studied. The percentage of trees in which the associated taxa clustered together is shown next to the branches (only values above 50 are shown).

The double BLAST search (www.ncbi.nlm.nih.gov accessed on 28 February 2024) using the 1.2 kbp of ribosomal RNA operon of these sequences showed that tamarisk phytoplasma strain with GenBank accession number MK313753 (strain Taa83) had 99.52% identity to ‘*Ca*. P. asteris’ with three GAPs and three SNPs to the reference strain (GenBank accession number M30790). The sequence having GenBank accession number MK313752 (strain Taa70=82) presented 99.84% identity to ‘*Ca*. P. australasiae=australasiaticum’ (GenBank accession number Y10097) with one GAP and one SNP to it, and the sequence in GenBank under the accession number of MK313754 (strain Taa84) had 99.60% identity to ‘*Ca*. P. trifolii’ with five SNPs to it. The phylogenetic trees generated by the analysis of 1.2 kbp 16S rRNA genes confirmed the information showing the clustering with the respective ‘*Ca*. Phytoplasma’ species. The phylogenetic trees constructed with phytoplasmas in subgroups of 16SrI, 16SrII, and 16SrVI and the representatives of TWB strains confirmed that these phytoplasma strains are close to ‘*Ca*. P. asteris’, ‘*Ca*. P. australasiae=australasiaticum’, and ‘*Ca*. P. trifolii’, respectively (Figure 5). 

**Figure 6:**
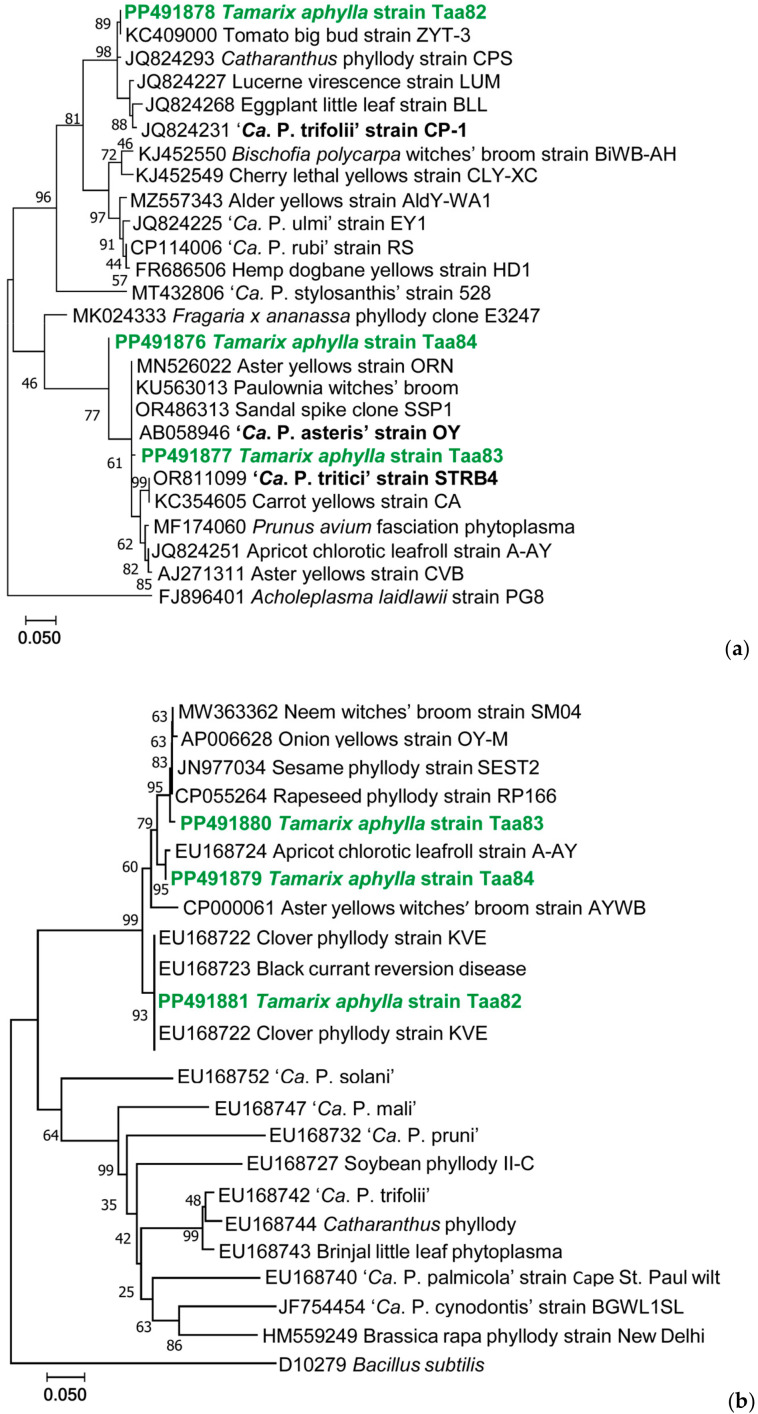
Molecular phylogenetic analysis by Maximum Likelihood method of the sequences of *tuf* (**a**) and *secA* (**b**) genes of *T. aphylla* phytoplasmas (in bold green) with different ‘*Ca*. Phytoplasma’, in bold ‘*Ca*. Phytoplasma’ reference strains; *Acholeplasma laidlawii* and *Bacillus subtilis* sequences are used as outgroups. Numbers at the nodes are bootstrap values based on 1000 repetitions. GenBank accession numbers for sequences are given before the phytoplasma name. The percentage of trees in which the associated taxa clustered together is shown next to the branches.

Three of the samples in which each of the three diverse phytoplasmas were detected (Taa82, Taa83, and Taa84) were employed for further amplification on *tuf* and *secA* genes, and the amplicons were sequenced, aligned, and used for phylogenetic tree preparation (Figure 6). The results showed consistency with results obtained for the 16S rRNA gene only for one of the samples, resulting in only sample Taa83 being infected with a ‘*Ca*. P. asteris’ strain (Table 1). The phytoplasmas detected in sample Taa84 showed ‘*Ca*. P. trifolii’ in the 16S rRNA gene and a ‘*Ca*. P. asteris’ strain in the other two genes, while the sample Taa82 showed an infection by three diverse ‘*Ca*. Phytoplasma’ species.

## 3. Discussion

Chah Afzal area’s planted forestry of *T. aphylla* was established in the 1980s in a 4000-hectare land as an anti-desertification effort. Over time, the cultivation of crops has also increased in the vicinity of this forest. More than a decade ago, in rural areas of central regions of Iran, suspected phytoplasma disease symptoms were observed for the first time in planted *T. aphylla,* used as forest and field’s windbreak. So far, up to 72% presence of witches’ broom disease was confirmed in planted tamarisk. The present survey indicates the presence of diverse phytoplasmas in tamarisk with witches’ broom and yellowing symptoms. Based on RFLP and phylogenetic analysis on 16S rRNA gene, the presence of 16SrI-B, 16SrII-D, and 16SrVI-A phytoplasma strains, i.e., ‘*Ca*. P. asteris’, ‘*Ca*. P. australasiae=australasiaticum’ [8] and ‘*Ca*. P. trifolii’, was detected in the symptomatic tamarisk plants. Moreover, the further amplification of single copy housekeeping gene, widely employed for phytoplasma detection and molecular characterization, indicated the presence of mixed phytoplasma infection as confirmed by the re-amplification of the same samples in which the above phytoplasmas were identified. This is among the first reports in which different phytoplasmas could be detected in the same samples using different phytoplasma gene targets. A case of similar mixed infection was recently reported in India in neem plants showing witches’ broom symptoms after the re-amplification of samples on *secA* gene [9]. This represents also the first characterization of these phytoplasmas in tamarisk showing witches’ broom and yellowing in Iran and worldwide. 

The phytoplasmas identified in symptomatic tamarisk trees were detected in a wide range of agricultural, forest, and rangeland plant species in Iran. Among the numerous phytoplasma strains detected in the Iranian flora, plant species reported as hosts of ‘*Ca*. P. asteris’ strains included *Eucalyptus camaldulensis* [10], ], while described as host plants for ‘*Ca*. P. australasiae=australasiaticum’ are *Sorghum halepense* [11], *Punica granatum* [12], *Sesamum indicum* [13], and *Calendula officinalis* [14]. *Brassica oleracea* and *B. napus* [15,16], *Lycopersicum esculentum* [17], and *Erigeron canadensis* [11], are reported as hosts of ‘*Ca*. P. trifolii’, while *Medicago sativa* and *Cucumis sativus* were infected by both the latter ‘*Ca*. Phytoplasma’ species, among others [18,19,20]. Moreover, *S. indicum* and *C. officinalis* showing phyllody, and *M. sativa* with witches’ broom were detected to be infected with ‘*Ca*. P. australasiae=austalasiaticum’ [13,14,18] and were grown in Chah Afzal next to tamarisk plants infected with witches’ broom. *T. aphylla* may have therefore a key role in the epidemiology of phytoplasma diseases as a reservoir species in a threat to agricultural, forest, and rangeland plant species in Iran. 

*Orosius albicinctus* is an important leafhopper species that transmits 16SrII phytoplasma strains and *T. aphylla* adjacent to agricultural fields is a reservoir site for this leafhopper [21]. Different nymphal stages of *O. albicinctus* survived on *T. aphylla* in the winter and the summer. Alfalfa witches’ broom, vectored by *O. albicinctus*, is the most important disease of *M. sativa* in central areas of Iran where up to 100% of disease incidence was observed. It is possible that the leafhopper vector transfers the phytoplasmas from infected tamarisk tree to other plant species in winter days with moderate temperature. Formerly, witches’ broom, yellowing, and decline due to 16SrI and 16SrII phytoplasma strains were reported from *Pistachia vera* fields in Iran [22]. The occurrence of symptomatic *T. aphylla* infected with 16SrI and -II phytoplasma strains adjacent to these farms in the Chah Afzal area can be a potential threat to pistachio cultivation. 

## 4. Materials and Methods

Among the five surveyed areas of the central regions in Iran, the occurrence of tamarisk phytoplasma disease was observed only in Chah Afzal, Mileshbar, and Bafruiyeh areas (Figure 1). In each area, sampling was carried out randomly in five plots, each of one hectare of planted tamarisk. A total of 150 samples (25 samples per each area) with symptoms were collected for testing. Twelve samples (two per area) were collected as a negative control from symptomless trees. To estimate the disease presence in each area, the total number of symptomatic plants was divided by the total number of plants in the sampling areas. The value obtained was expressed as a percentage by multiplying it by 100. 

All sampled plants were propagated by branch cutting for dodder inoculation to periwinkle, and molecular studies and the assessment of growth conditions were performed. For the propagation of tamarisk plants, 25 cm branch cutting was taken from trees showing symptoms of witches’ broom and yellowing and also from symptomless trees as a control. All tamarisk branch cuttings were planted in soil pots and kept in an insect-free greenhouse under 27 °C and 50% of relative humidity for rooting. The date of sprouting, the sprouting rate, and the seedling height of cuttings were measured. 

Transmission of phytoplasmas via dodder to periwinkle (*Catharanthus roseus*) was carried out. Seeds of dodder (*Cuscuta campestris*) were germinated on moist filter paper, and seedlings were transferred to two-month-old tamarisk sprouted branch cuttings under insect-proof conditions. After six weeks, newly developed strands of dodder from infected tamarisk seedlings were connected to seed-grown healthy periwinkle at 4–5 leaf stage. The connection was maintained for 45 days, and then, dodder-inoculated periwinkles were kept in an insect-proof greenhouse for the evaluation of symptom expression. Negative controls were prepared by the inoculation of ten periwinkle plants by dodder strands grown on symptomless tamarisk. 

Total DNA was extracted from 0.2 g of *T. aphylla* leaves with witches’ broom and yellowing branches and from asymptomatic tamarisk using the methods described by Healey et al. [23] based on CTAB and chloroform/phenol methods. Total DNA extracted from *Calendula officinalis* phyllody phytoplasma (16SrII-D) [14] was used as a positive control. A total of 100 ng of nucleic acid from symptomatic and symptomless tamarisk samples and from the control phytoplasma was used for nested polymerase chain reaction using P1/P7 [24,25], R16mF2/R16mR2, and R16F2n/R16R2 [26] primer pairs in PCR and nested PCR, respectively. The nested PCR reactions were performed using the 1: 30 dilutions of amplicons obtained from PCR as a template. The amplifications were carried out in a programmable thermocycler (Bio-Rad, Hercules, CA, USA) following described conditions [27]. PCR products were electrophoresed in 1% agarose gel in the TBE buffer and visualized with a UV transilluminator following ethidium bromide staining. The molecular weight of the PCR products was estimated by comparison with 100 bp DNA ladder (Biobasic, Markham, ON, Canada).

RFLP analyses were carried out on the 1.2 kb nested PCR products from R16F2n/R16R2 primer pairs using each of the restriction enzymes, *Mse*I, *Hh*aI, *Alu*I, *Hae*III, *Rsa*I, *Hpa*II, *Hpa*I, *Taq*I, *Kpn*I, *Sau3A*I, *Tha*I, and *Bfa*I, separately according to the instructions of the manufacturer (Thermo, Vilnius, Lithuania). The restriction products were then separated by electrophoresis through a polyacrylamide 6.7% gel stained with ethidium bromide and visualized with a UV transilluminator. The resulting RFLP patterns were compared with those previously published for 16S rDNA of other phytoplasmas [5]. Virtual RFLP analysis using *i*PhyClassifier [6] was used to verify the subgroup affiliation of tamarisk phytoplasma strains comparing the sequences of a representative strain of each group to those of available phytoplasmas in groups 16SrI, 16SrII, and 16SrVI. Selected samples in which diverse phytoplasmas were identified on the 16S rRNA gene were employed for nested PCR amplification on *tuf* and *secA* genes using reported primers and conditions [28,29]. 

The R16mF2/R16mR2 primed PCR products of six samples from each of the determined phytoplasma subgroups and the *tuf* and *secA* genes amplicons from three of the same samples in which the diverse phytoplasmas were detected and were directly sequenced in both directions using M13F and T7 [28] for the *tuf* gene and SecAfor5/SecArev2 for the *secA* gene [30], respectively. The assembled sequences were compared with deposited sequences in Gene Bank database using Blast (National Center for Biotechnology Information, Bethesda, MD, USA) and aligned with BioEdit software 7.0.5.3. The evolutionary history was inferred by using the Maximum Likelihood method based on the Jukes–Cantor model [31]. Initial tree(s) for the heuristic search were obtained automatically by applying Neighbor-Join and BioNJ algorithms to a matrix of pairwise distances estimated using the Maximum Composite Likelihood (MCL) approach, and then by selecting the topology with the superior log likelihood value. Phylogenetic trees were generated using MEGA 7 software [32]. *Acholeplasma laidlawii* and *Bacillus subtilis* were used as an outgroup to root the trees. Bootstrapping was performed 1000 times to estimate the clade stability and support for the branches.

## 5. Conclusions

In plant infectious disease epidemiology, the co-presence of pathogens, susceptible host, and the possible natural transmission especially by insects is important. Tamarisk trees that were planted over the past decades for anti-desertification programs and as windbreaks in Iran become infected with different phytoplasma strains determining a major threat to all agricultural and natural resources in the areas. It is necessary to prevent the disease spread as it is proved according to the results of this work that the tamarisk trees planted for anti-desertification are infected with diverse phytoplasmas transmitted from other plant species already present at the time in the environment, or the propagation material could have been already infected them in nurseries. This was aggravated when, during drought periods, the phytoplasma insect vectors transmitted phytoplasma strains to the tamarisk trees during insect over-wintering and over-summering. The presence of mixed phytoplasma infection in the symptomatic tamarisk trees indicated that tamarisk trees are both good and sensitive hosts for being infected by phytoplasmas and play an essential role in the spread of the disease to other plants.

## Figures and Tables

**Table 1:** Summary of results of amplification on 16S rRNA and *tuf* and *secA* genes for phytoplasma identification.Nd, not determined.

*Tamarix aphylla* Sample	16S rRNA Gene (Ribosomal Group) GenBank Accession Number	tuf Gene (Ribosomal Group) GenBank Accession Number	secA Gene (Ribosomal Group) GenBank Accession Number
Taa 70=82	‘*Ca*. P. australasiae=australasiaticum’ (16SrII-D) MK313752	‘*Ca*. P. trifolii’ (16SrVI-A) PP491878	‘*Ca*. P. graminis’ (16SrI-C) PP491881
Taa83	‘*Ca*. P. asteris’ (16SrI-B) MK313753	‘*Ca*. P. asteris’ (16SrI-B) PP491877	‘*Ca*. P. asteris’ (16SrI-B) PP491880
Taa84	‘*Ca*. P. trifolii’ (16SrVI-A) MK313754	‘*Ca*. P. asteris’ (Nd) PP491876	‘*Ca*. P. asteris’ (16SrI-F) PP491879

## Data Availability

The original data presented in the study are openly available at GenBank under the accession numbers listed in the paper.
